# Phenotypic and Immunomodulatory Properties of Equine Cord Blood-Derived Mesenchymal Stromal Cells

**DOI:** 10.1371/journal.pone.0122954

**Published:** 2015-04-22

**Authors:** Laurence Tessier, Dorothee Bienzle, Lynn B. Williams, Thomas G. Koch

**Affiliations:** 1 Department of Biomedical Sciences, Ontario Veterinary College, University of Guelph, Guelph, Canada; 2 Department of Pathobiology, Ontario Veterinary College, University of Guelph, Guelph, Canada; 3 Department of Clinical Studies, Ontario Veterinary College, University of Guelph, Guelph, Canada; 4 The Orthopaedic Research Lab, Aarhus University, Aarhus, Denmark; French Blood Institute, FRANCE

## Abstract

Multipotent mesenchymal stromal cells (MSC) have attracted interest for their cytotherapeutic potential, partly due to their immunomodulatory abilities. The aim of this study was to test the robustness of our equine cord blood (CB) MSC isolation protocol, to characterize the CB-MSC before and after cryopreservation, and to evaluate their immunosuppressive phenotype. We hypothesized that MSC can be consistently isolated from equine CB, have unique and reproducible marker expression and *in vitro* suppress lymphoproliferation. Preliminary investigation of constitutive cytoplasmic Toll-like receptor (TLR) 3 and 4 expression was also preformed due to their possible association with anti- or pro-inflammatory MSC phenotypes, respectively. Surface markers were assessed for antigen and mRNA expression by flow cytometry and quantitative polymerase chain reaction (qPCR). Immunomodulatory properties were evaluated in mixed lymphocyte reaction assays, and TLR3 and TLR4 expression were measured by qPCR and immunocytochemistry (ICC). CB-MSC were isolated from each off nine cord blood samples. CB-MSC highly expressed CD29, CD44, CD90, and lacked or had low expression of major histocompatibility complex (MHC) class I, MHC-II, CD4, CD8, CD11a/18 and CD73 before and after cryopreservation. CB-MSC suppressed *in vitro* lymphoproliferation and constitutively expressed TLR4. Our findings confirmed CB as a reliable MSC source, provides an association of surface marker phenotype and mRNA expression and suggest anti-inflammatory properties of CB-MSC. The relationship between TLRs and lymphocyte function warrants further investigation.

## Introduction

Cell-based therapies are increasingly in demand for treatment of a variety of conditions, including equine osteoarthritis [1]. However, our understanding of stem and stromal cell properties is evolving slower than clinical applications are being pursued. The use of variably characterized stromal cell preparations has led to discrepancies between predicted and observed efficacy, and between different studies [1–3]. Development of safe and efficacious cell-based treatments is crucial for clinical application and to define potential value as novel therapy.

Multipotent mesenchymal stromal cells (MSC) are potential candidates for cell-based therapy [2,4–6]. These cells are most commonly derived or isolated from bone marrow (BM), adipose tissue (AT) or umbilical cord blood (CB). In human research, the term MSC is often associated with mesenchymal stem cells rather than mesenchymal stromal cells. Here, we exclusively refer to mesenchymal stromal cells. Stem cells are characterized by long-term self-renewal and differentiation abilities [7]. In horses, MSC differentiation abilities are still poorly understood, and no evidence of *in vivo* long-term self-renewal ability have been published. Therefore, mesenchymal stem cells remain uncharacterized in this species, precluding their reference in the present paper. In humans, MSC are evaluated based on minimal classification criteria that were established by the International Society for Cellular Therapy (ISCT). Criteria include plastic adherence, osteo-, chondro- and adipogenic differentiation, and cell surface expression of CD73, CD90, and CD105 concurrent with absent expression of CD11b or CD14, CD45, CD34, CD79a or CD19, and human leukocyte antigen (HLA)-DR [8]. However, MSC remain incompletely characterized, and the above marker panel is largely exclusive rather than inclusive. MSC derived from animals are less well defined, and may also differ from human MSC. Therefore, improved and consistent culture methods for MSC and more comprehensive phenotypic characterization are required.

Equine MSC are less well characterized than human MSC, and inconsistent surface marker profiles have been observed. Surface expression of CD29, CD44 and CD90 was reported in several studies. However, unlike for human MSC, variable identification of CD73 and CD105 on MSC has precluded establishment of a consensus panel for horse MSC [9–17]. In addition, several investigators reported expression of MHC-II, CD31, CD34, CD45 and CD79a to be low or absent [9–28]. Hence, surface antigens characteristic of equine MSC have neither been clearly established nor confirmed with quantitative gene expression assays, although clinical application of such cells has been widely implemented [6]. Furthermore, MSC are often cryopreserved for potential future clinical use. Phenotypic and functional stability during such storage is unknown.

MSC progenitor function is defined by ability to differentiate into multiple cell types including osteo-, chondro- or adipogenic tissue [8]. MSC were also suggested to influence proximal cells *in vivo* by secreting trophic and immunomodulatory factors [29]; a non-progenitor cell function that attracted much attention due to potential for also treating conditions associated with aberrant immune responses. *In vivo* immunosuppressive function was reported for human MSC that were used for successful treatment of steroid-resistant stage IV graft-versus-host disease (GVHD) [30]. Promising results for treatment of other inflammatory diseases, such as osteoarthritis, with MSC has also been reported for several species [6,31]. Analysis of short-term outcomes indicated reduced inflammatory cytokine production and enhanced cartilage regeneration. *In vitro* anti-proliferative effect of equine MSC on lymphocyte proliferation was reported [23]. However, results from *in vitro* and *in vivo* studies are inconsistent, and evidence of long-term therapeutic efficacy is lacking [6,32].

The phenotype of MSC with immunomodulatory capability is unknown, which precludes derivation of cell populations for such purpose. It has been suggested that MSC, similarly to macrophages, might include MSC-1 and -2 subpopulations with pro-inflammatory and anti-inflammatory properties, respectively. MSC-1 and -2 subpopulations have been hypothesized to be associated with expression of TLR4 or TLR3, respectively [33]. MSC from different species or tissues expressed distinct TLR transcripts and antigens [34–36], suggesting functional properties might be linked to tissue origin. Equine CB-MSC suppressed lymphoproliferation *in vitro* [23], but correlation with TLR3 or TLR4 expression was not determined.

The aims of this study were to determine the efficacy of deriving MSC from equine CB, to determine phenotype before and after cryopreservation, to evaluate whether CB-MSC suppress lymphocyte proliferation, and to assess expression of TLR3 and TLR4. We report highly efficient derivation of MSC with consistent phenotype from equine CB. Phenotypic and functional characteristics were preserved through cryostorage and associated with expression of TLR4.

## Materials and Methods

### Ethics statement

This study was specifically approved by the University of Guelph Animal Care Committee with regard to the procedures of collection of equine peripheral blood lymphocytes and equine umbilical cord blood (animal use protocols 1756 and 1570). Additional research conducted using specimens of this kind does not require review by the Animal Care Committee (falls under CCAC Category of Invasiveness A) and therefore the mixed lymphocyte reactions can be considered to have been conducted in accordance with the institutional ethics guidelines. Collection of peripheral blood and cord blood was add-on procedures to the routine care of the horses. No animals were sacrificed during the study. Equine umbilical cord blood was collected on two privately owned commercial farms in Southern Ontario. Four of five samples were collected on one farm from Thoroughbred foals. One sample was collected on another farm from a Warmblood foal. Informed consent was obtained in writing from the horse owners/agents prior to sampling. All the data for this study was subsequently anonymized. The broodmares on the foaling farms are housed in large foaling boxes. Both farms are staffed 24/7 and mares are under constant video surveillance and carrying foaling alarms to allow for observed foaling and assisted delivery if needed. Umbilical cord blood was collected by the farm staff after receiving instruction by Dr. Koch. Instruction included video-review of cord blood collection. Cord blood was collected from an isolated segment of the umbilical cord after the umbilical cord had been clamped and detached from the foal. Peripheral venous blood was obtained from the Equine Research Herd owned by the Ontario Ministry of Agriculture and Food (OMAF). Once investigators have an approved animal care protocol from the University of Guelph Animal Care Committee access to these research horses are granted. In this study peripheral blood was collected from 5 adult mixed-bred horses. The adult horses on the research farm are housed in smaller groups with run-in sheds throughout the year. The horses are on pasture during the summer and during the winter they have access to large paddocks with gravel surface. Peripheral blood was collected by Drs. Williams or Koch. Peripheral blood was collected under mild sedation (Xylazine HCl, 0.35–0.40 mg/kg bwt IV; Bayer, Toronto, ON) from the jugular vein following which manual pressure was applied for several minutes to aid hemostasis. All the data for this study was subsequently anonymized. This study was also specifically approved by the University of Guelph Research Ethics Board with regard to the procedures of collection of Human peripheral blood. Human peripheral blood was collected in agreement with Institutional Research Ethics Board guidelines (protocol 12FE008). Informed written consent was obtained from the participants.

### Experimental design

**Fig 1:**
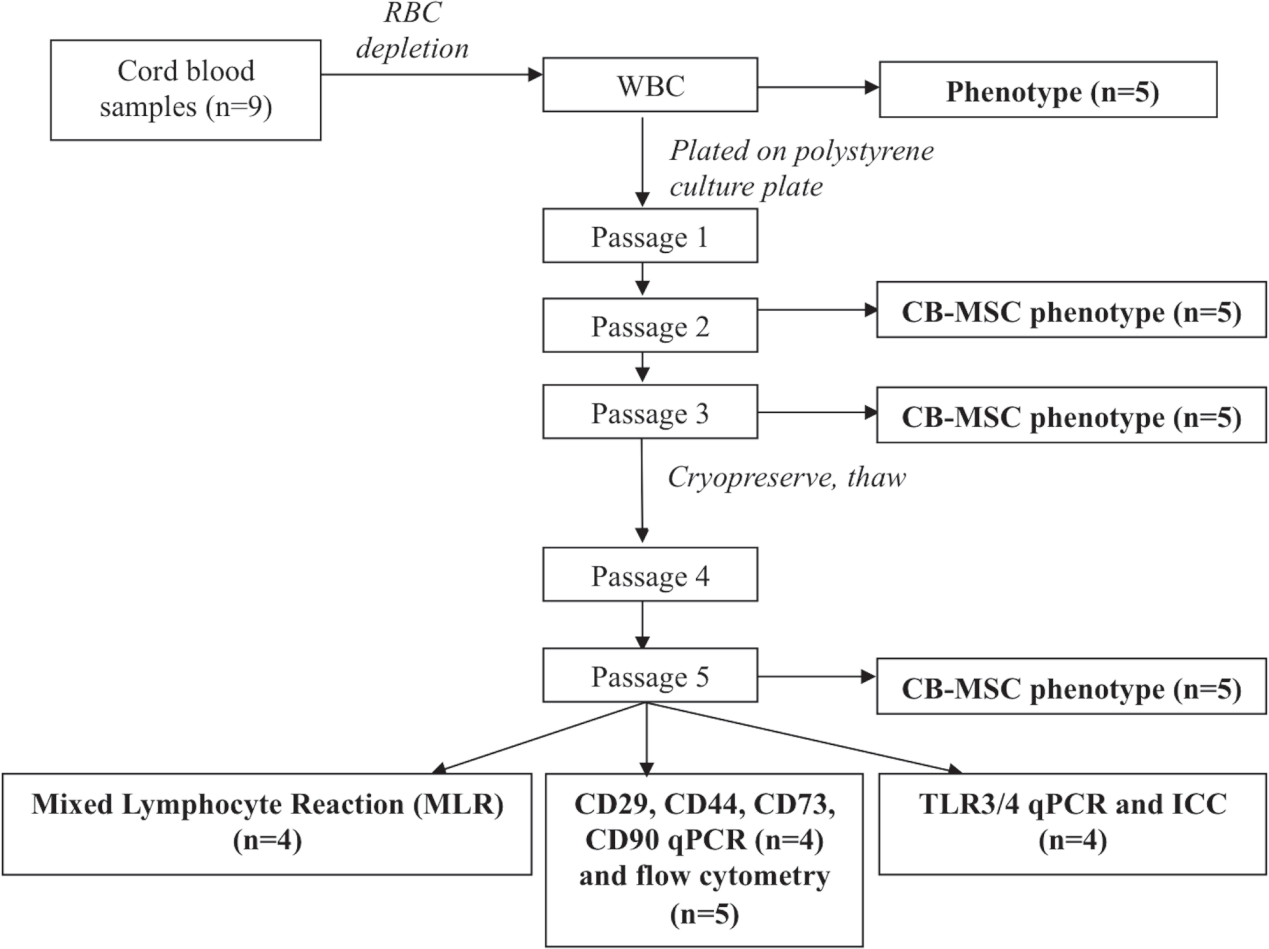
Schematic of experimental design.

CB-MSC cultures were established from nine individual samples and analyzed for phenotype, mRNA expression and functional properties, as outlined (Fig 1).

### CB collection and shipping

CB was collected from nine foals immediately after foaling, as previously described [37]. CB collection was approved by the Institutional Animal Care Committee (protocol 1570). Venipuncture of the umbilical vein was performed with a 16G hypodermic needle attached to a 450 mL blood transfusion collection bag (Fenwal, Baxter, Deerfield, IL) containing citrate phosphate dextrose adenine as the anticoagulant solution. The blood was stored at 4–8°C for up to 24 hours before and up to 12 hours after being transported overnight by courier. A Greenbox thermocontrol device (Greenbox System, ThermoSafe Brands; Arlington Heights, VA) was used for shipping and temperature control for up to 48 hours to account for possible delay during shipping. No samples encountered shipping delay.

### CB-MSC isolation and culture

CB-MSC isolation and culture were adapted from a previously described protocols [38]. In brief, the white blood cell (WBC) fraction was isolated using PrepaCyte-WBC (PC, CytoMedical Design Group; St. Paul, MN). Whole CB was mixed 1:1 with PC medium in 50 mL BD Falcon conical tubes (BD Biosciences, Mississauga, ON) for 5 minutes, and then incubated for 25 minutes at room temperature (RT). Supernatant was collected, pooled and spun at 400*g* for 10 minutes at RT. Pellets were suspended in isolation medium (IM) consisting of Dulbecco’s modified eagle medium (DMEM)-low glucose (1 g/L; Lonza, Wakersville, MD), 30% fetal bovine serum (FBS; Invitrogen, Burlington, ON), penicillin (100 IU/mL; Invitrogen), streptomycin (0.1 mg/mL; Invitrogen), L-glutamine (2 mM; Sigma-Aldrich, St. Louis, MO), and dexamethasone (10^-7^M; Sigma-Aldrich). Cells were then incubated at 5% CO_2_ at 38°C in humidified atmosphere. Live and dead cells were counted with an automated cell counter (NucleoCounter NC-100, Mandel Scientific, Guelph, ON), and plated at 1x10^6^/cm^2^ live cells in 75 cm² polystyrene cell culture flasks (Corning, Sigma-Aldrich). Primary colonies were detached using trypsin/ethylene diamine tetraacetic acid (EDTA; 0.04%/0.03%; Sigma-Aldrich) and hereafter expanded in the same culture medium without dexamethasone (expansion medium, EM). During expansion, cells were seeded at 5,000/cm^2^. Cells were cryopreserved in EM containing 10% dimethyl sulfoxide (DMSO; Sigma-Aldrich) at -1°C/min controlled freezing rate to -80°C for 24 hours, prior to long-term storage in liquid nitrogen. Cell concentration in the freezing medium was 1x10^6^/mL.

### Cell morphology, colony counting, CB-MSC progenitor frequency, and population doubling time

After eight days of incubation, cultures were inspected daily for presence of colonies. Confluent colonies were detached using trypsin/EDTA and re-seeded in T175 culture flasks. To document cell morphology, digital images were obtained prior to detachment at passage 1, 3, 4 and post-thawing passage 5 using phase-contrast microscopy and Q-Capture software (Q-Imaging, Surrey, BC). CB-MSC progenitor frequency (PF) and doubling times were calculated as:

*PF* = *Colony number* / *Number of WBC x* 100

Doubling times were calculated according to:

1. *cell–doubling number(CD) = In(Nh/Ns)/In*2
  - *In* = *natural Logarithm*
  - *Nh* = *harvest cell number*
  - *Ns* = *seed cell number*
2. *Cell doubling time (DT)* = *CT/CD*
  - *CT* = *cell culture time*

### Flow cytometry

The WBC fraction in CB samples, and CB-MSC at passage 2, 3 and 5, were analyzed by flow cytometry in a FACScan instrument (BD, Mississauga, ON) with a panel of antibodies (Table 1). Antibodies for CD29, CD44, CD73 and MHC-I clone CVS22 were acquired later in time and were tested only on CB-MSC at passage 5. Reactivity with positive control samples was first assessed. Antibodies and their positive control(s) are listed in Table 1. All antibodies were titrated for optimal dilution to label positive control cells. Since CD73 clone 10f1 antibody did not react with equine CB-MSC, antibody was assessed and confirmed for reactivity with human (antibody target specie) and equine epitopes using human and equine WBC respectively. CD73 has a nucleotide identity of 88% between horses and humans and staining of human and equine WBC was 18% and 5% respectively (data not shown). Human peripheral blood was collected in agreement with Institutional Research Ethics Board guidelines (protocol 12FE008), and blood samples from horses according to an approved animal care protocol (#11R034). In addition, CD73 clone 10f1 was reported to cross-react with equine cells using flow cytometry and confirmed with confocal microscopy [16]. All other antibodies used in this study were tested on equine WBC as part of the feasibility studies (data not shown). Incubations were at 4°C in the dark for 15 minutes, followed by a wash, and secondary antibody incubation at 4°C for 15 minutes in the dark. Rat anti-mouse IgM-FITC, goat anti-mouse IgG1-FITC (both Abcam, Toronto, ON) or donkey anti-mouse IgG (H+L)-FITC (Jackson ImmunoResearch Laboratories Inc., West Grove, PA) were used as secondary antibodies. Prior to staining, ammonium chloride (Sigma-Aldrich) hypertonic red blood cell (RBC) lysis was performed on peripheral and CB for leukocyte isolation, followed by a wash with flow buffer ((phosphate buffer saline (PBS); Sigma Aldrich), 5mM EDTA, 1% horse serum (HS), and 0.1% sodium azide). Cultured CB-MSC were chemically detached with Accumax (Stemcell Technologies Inc., Vancouver, BC) and washed with flow buffer prior to antibody incubation. WBC and CB-MSC at passage 2, 3 and 5, were assessed for CD4, CD8, CD11a/18, CD90, MHC-I and MHC-II surface expression, and CB-MSC at passage 5 for CD29, CD44 and CD73 expression. Negative control samples were cells incubated with only secondary antibody, and with isotype-matched non-binding primary antibody plus fluorescent secondary antibody. A minimum of 10,000 events were acquired for each antibody with CellQuest software (BD) and analyzed with FlowJo software (Tree Star Inc., Ashland, OR). Gates to identify WBC or CB-MSC populations were maintained consistent throughout all experiments.

**Table 1:** Antibody panel for flow cytometric analysis.

Antigen	Source	Clone	Positive control samples
CD4	abdSerotec^a^	CVS4	Equine WBC
CD8	abdSerotec	HTI4A	Equine WBC
CD11a/18	abdSerotec	116.2D11B10	Equine WBC
CD29	Beckman Coulter^b^	4B4	Equine MSC
CD44	abdSerotec	CVS18	Equine WBC
CD45	VMRD^c^	DH16A	Equine WBC
CD73	Abcam	10f1	Human & equine WBC
CD90	VMRD	DH24A	Equine WBC
MHC-I	abdSerotec	117.1B12C11	Equine WBC
MHC-I	abdSerotec	CVS22	Equine WBC
MHC-II	abdSerotec	130.8E8C4	Equine WBC

^a^ Raleigh, NC

^b^ Mississauga, ON

^c^ Pullman, WA

### Quantitative PCR

Total RNA was extracted from WBC and CB-MSC at passage 3, 4, and 5 using the *mir*Vana miRNA Isolation Kit (Ambion, Life Technologies, Burlington, ON) following the manufacturer’s instructions. RNA was quantified using a spectrophotometer (**NanoDrop ND-1000**, Thermo Fisher Scientific, Waltham, MA), aliquoted, and stored at -80°C for reverse transcription. For complementary DNA (cDNA) synthesis, RNA was thawed, and any potential residual genomic DNA was digested using DNase I amplification grade treatment (Invitrogen), directly followed by reverse transcription using the SuperScript II Reverse Transcriptase kit (Invitrogen) with random priming of RNA. RNA quality was assessed by capillary electrophoresis (**Bioanalyzer 2100**, *Agilent* Technologies, Mississauga, ON) and only samples with RNA integrity numbers (RIN) above 9 were used.

Relative quantification was performed in a CFX Real-Time PCR Detection System (Bio-Rad, Kitchener, ON) by two-step real-time qPCR. Primers (Table 2) were designed using Invitrogen OligoPerfect Designer or the *National Center for Biotechnology Information* (NCBI) primer-BLAST software, or adapted from previously published sequences [39]. Beta-2-microglobulin (B2M) and class II MHC transactivator (CIITA) sequences were used to detect MHC-I and-II, respectively. Amplicons were separated by 2% agarose gel electrophoresis, extracted and purified using the QIAquick Gel Extraction kit (Qiagen, Hilden, Germany). DNA was sequenced and sequences were analyzed using the NCBI basic local alignment search tool (BLAST) against the equine database. Minimal identity cut-off was set at 96%. All primers except those for CD4 and CD73 incorporated at least one intron-exon spanning junction. To ensure specificity of all primers, sample without reverse transcription (RT-control) and samples lacking cDNA template (NT-control) were included in each run. PCR assays were performed in triplicate in a 10 μL total volume consisting of 5 μL of PCR SsoFast EvaGreen Supermix (Bio-Rad), 0.2 μM of primer mix and cDNA template. After an initial incubation at 95°C for 3 min, reactions were cycled 40 times with denaturation at 95 °C for 5s, annealing for 5s at temperatures specific for each primer pair, and extension at 72 °C for 15s. Amplification specificity was determined with melting-curve analysis whereby each amplicon was heated from 65 to 95°C. Efficiency for each primer pair was calculated from a standard curve generated from cDNA of leukocytes or CB-MSC. qPCR data were analyzed by the delta-delta Ct method (∆∆Ct) [40] using two endogenous reference genes chosen following the minimum information for publication of quantitative real-time PCR experiment (MIQE) guidelines [41]. Expression stability analysis of the reference genes was performed with geNorm software. Initial selection of housekeeping genes was based on review of current literature [14,42], and comprised β-actin, S18, B2M, and hypoxanthine phosphoribosyltransferase 1 (HPRT1). Based on geNorm [43] analysis of minimal variability in expression across samples, two housekeeping genes were selected. Assessment of variability in expression of different genes was based on the M-value calculated as the average pairwise variation of a gene compared to the other housekeeping genes [44]. The initial cut-off M-value was <1.5, and S18 and β-actin were selected to be used together as reference genes for subsequent relative quantification.

**Table 2:** Nucleotide sequence of primers.

Gene	Primers
CD4	5′ CCAGACTGACCAGACTGCAA 3′
	5′ TTGGATTCCAGCAGGACTTT 3′
CD8	5′ AGTGGCTGGACTTCGACTGT 3′
	5′ CAAACACGTCTTCGGTTCCT 3′
CD11a/18	5′ TTCAGCCAGCAACAAGAAGA 3′
	5′ GACAGCTGTGTTCCCACTGA 3′
CD29	5′ CCCTTGCACAAGTGAACAGA 3′
	5′ ATTCCTCCAGCCAATCAATG 3′
CD44	5′ ATCCTCACGTCCAACACCTC 3′
	5′ CTCGCCTTTCTTGGTGTAGC 3′
CD73	5′ TGATCTTTCCCGAAAACCTG 3′
	5′ GGAATCCATCTCCACCATTG 3′
CD90	5′ TGCCTGAGCACACATACCGCTC 3′
	5′ GCTTATGCCCTCGCACTTGACC 3′
B2M^a^	5′ TCGGGCTACTCTCCCTGACT 3′
	5′ ATTTCAATCTCAGGCGGATG 3′
CIITA^b^	5′ GGTGCTACTTCGAGCTTTCG 3′
	5′ CCAACGTAGAGTCCGGTGAG 3′
TLR4	5′ CCCACATCAACCAAGGAACT 3′
	5′ ATGGTTGAGGCCCTGATATG 3′
TLR3^c^	5′ CAAACCCTGGTGGTCCTGTT 3′
	5′ GAAGGCCTCTGCTGGGATCT 3′
β -actin	5′ TGGGCCAGAAGGACTCATAC 3′
	5′ GGGGTGTTGAAGGTCTCAAA 3′
S18	5′ ACTGAGGATGAGGTGGAACG 3′
	5′ GCCCGTATCTTCTTCAGTCG 3′

^a^ B2M sequence was used for gene expression detection of MHC-I

^b^ CIITA sequence was used for gene expression detection of MHC-II

^c^ Previously published sequence [39].

### Lymphocyte proliferation assay

Blood was obtained from the jugular vein of five adult horses of variable breed and sex with an 18G hypodermic needle attached to a 450 mL blood transfusion collection bag (Fenwal, Baxter, Deerfield, IL). Mononuclear cells (MNC) were isolated a using Ficoll-density gradient. At RT, 15 mL of Ficoll-Paque Plus (density 1.078 g/mL, Stemcell Technologies) was loaded in a 50 mL tube with 35 mL of whole blood. Gradients were centrifuged at 300*g* for 30 min at RT with no brake. Interphases containing the MNC fraction were carefully removed, pooled and washed with PBS. Supernatant was then removed and pellets were washed again with 10 mL of PBS. Pellets were then resuspended in 10 mL of Roswell Park Memorial Institute (RMPI) 1640 medium supplemented with penicillin (100 IU/mL; Invitrogen), streptomycin (0.1 mg/mL; Invitrogen) and 10% FBS (Invitrogen), and a live cell count was performed with an automatic cell counter (NucleoCounter NC-100). Cells were resuspended in freshly prepared cryomedium consisting of RPMI-1640 medium supplemented with penicillin (100 IU/mL; Invitrogen), streptomycin (0.1 mg/mL; Invitrogen), 10% FBS (Invitrogen) and 10% DMSO (Sigma-Aldrich) at a concentration of 6x10^6^ cells/mL. One mL of cell suspension was placed in a 2 mL cryovial (Corning, Sigma-Aldrich) on ice for 30 minutes before gradual freezing over 12–18 hours to -80°C, and storage in liquid nitrogen.

For assessing lymphocyte suppression, triplicate cultures of 1x10^5^ responder MNC were incubated with either 1x10^4^ irradiated autologous MNC or 1x10^4^ irradiated allogeneic MNC (pooled from three horses) in 96-well plates, yielding 10:1 responder:stimulator cell ratios. These cultures served as negative and positive controls, respectively. The test wells were comprised of 1x10^4^ irradiated CB-MSC, of 1x10^5^ MNC from horses unrelated to CB-MSC donors, and 1x10^4^ irradiated pooled allogeneic MNC (pooled from three horses unrelated to CB-MSC donors). Cells were cultured in RPMI 1640 media supplemented with L-glutamine (2 mM; Sigma-Aldrich), penicillin (100 IU/mL; Invitrogen), streptomycin (0.1 mg/mL; Invitrogen) and 10% heat inactivated horse serum (Invitrogen). Reactions were incubated for five days in round-bottom 96-well plates in 250–300 μL volume. Bromodeoxyuridine (BrdU) staining was performed with a FITC BrdU Flow Kit (BD Biosciences), and parameters for flow cytometry were set according to the manufacturer’s instructions. BrdU was added to MNC cultures on day five, and cells were incubated for 24 hours. Cells were then fixed and permeabilized, and FITC-conjugated antibody to BrdU was added for 20 minutes at RT. Subsequently, 7-aminoactinomycin D (7-AAD) was added for 5 minutes, and cells were analyzed with 5,000 events acquired per triplicate sample. Controls consisted of unstained cells and cells incubated with only FITC-conjugated antibody.

### TLR immunocytochemistry

MSC (5,000) were cultured in 250 μL of EM in 8-well Permanox chamber slides (Thermo Fisher Scientific) until 60–80% confluency. Cells were exposed to 1 μg/mL of polyinosinic:polycytidylic acid (poly I:C; Sigma-Aldrich) and 10 ng/mL of lipopolysaccharide (LPS; Sigma-Aldrich) for one hour to induce TLR3 and TLR4 expression. For ICC, cells were washed thrice with PBS, fixed *in situ* for 5 minutes with 4% paraformaldehyde (PFA; Invitrogen), washed thrice again with PBS and permeabilized with 0.1% Triton for 15 minutes. Cells were then washed again and treated with 3% hydrogen peroxide for 15 minutes. Non-specific antibody binding was blocked by incubation with 5% FBS for 10 minutes followed by one hour incubation with polyclonal primary antibody to TLR3 or TLR4 (both Imgenex, San Diego, CA). Cells were then washed thrice with PBS and incubated for one hour with goat anti-rabbit secondary antibody (Abcam). Bound antibody linked to horseradish peroxidase (HRP) was detected using 3,3'-diaminobenzidine chromogen (Dako, Burlington, ON). Cells were counterstained with Harris hematoxylin to visualize nuclei, and images were acquired on a phase contrast microscope.

### Statistical analysis

All data were analysed with statistical analysis software (SAS Institute, Cary, NC). Effects were considered significant at *P* < 0.05. Mixed lymphocyte reaction data and comparison between passages for flow cytometry data were analyzed using a three-factor ANOVA without replication with covariate (negative control). Comparison between markers for flow cytometry data were analyzed as single assays using a two-factor factorial ANOVA and Pmax as a covariate. Data were plotted as percent positive staining with 95% confidence intervals (CI). qPCR results was analyzed using a two-factor factorial in a randomized block with sub-sampling, accommodating unequal variances among targets to meet ANOVA assumption. Multiple comparisons were subsequently adjusted using Tukey’s honest significant difference (HSD) method. Data were plotted as relative expression with 95% CI. For CD8 and CIITA, transcripts were detected only in WBC, precluding comparisons. Analysis of residuals was performed to assess the ANOVA assumption. Residuals were plotted against the predicted values and explanatory variables used in the model. The residuals were tested for normality using the four tests offered by SAS: Shapiro-Wilk, Kolmogorov-Smirnov, Cramer-von Mises, and Anderson-Darling.

## Results

### MSC progenitor frequency, population doubling time, and cell morphology

All nine CB samples yielded colonies and subsequent CB-MSC cultures with classic spindle-shaped fibroblast-like morphology. Minimal differences were observed in the appearance of cells with increasing passage number and following cryopreservation with retention of fibroblast-like morphology. The number of colonies derived from each CB sample varied between 1 and 14, with a progenitor frequency of 1 progenitor per 2.25x10^8^ to 1.6x10^7^ WBC (average of 1 progenitor per 8.99 x10^7^ WBC). All CB-MSC cultures were expandable to yield 1.12x10^6^ to 1.37x10^7^ cells at the end of passage 2 (average of 4.70x10^6^ cells). Mean (± standard error of the mean (SEM)) doubling time (DT) in days were as follows: passage 2 to 3 (n = 9) 1.67 ± 0.15, passage 3 to 4 (n = 9) 1.68± 0.15, passage 4 to 5 after cryopreservation (n = 5) 1.89 ± 0.58. CB-MSC from all nine CB samples were expandable to allow cryopreservation of 1.42x10^6^ to 7.82x10^6^ cells after passage 3 (average of 3.8 x10^6^ cells).

### Loss of WBC markers and retention of CD90 in CB-MSC

**Fig 2:**
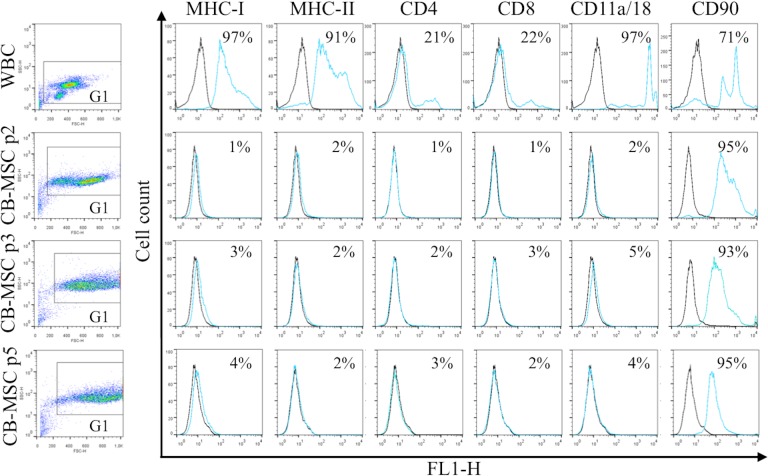
Antigen expression on WBC in CB, and CB-MSC at passage (p) 2, 3 and 5. Consistent gates were applied for all cell analyses. Control sample fluorescence is indicated by black lines, fluorescence of samples incubated with specific antibody by light blue/gray lines. Results were similar for samples from 5 animals. Numbers in each plot represent the mean proportion of cells expressing each antigen.

All antibodies yielded expected reactions except antibody to CD45 which did not react with equine cells. MHC-II was detected on WBC but not CB-MSC. Flow cytometric analysis (Fig 2) showed that the WBC fraction in CB contained cells that uniformly expressed MHC-I, MHC-II and CD11a/18, and variably frequent proportions of CD4^+^, CD8^+^, and CD90^+^ cells (Fig 2). With derivation of CB-MSC, expression of MHC-I, MHC-II, CD4, CD8 and CD11a/18 disappeared. A large proportion of WBC expressed CD90, which persisted throughout differentiation into CB-MSC. Statistical analysis revealed significant decreases in the proportion of cells expressing each marker except CD90. Changes in surface marker expression between the WBC fraction and the CB-MSC population at passage two was significant for all markers (p < 0.0001). No significant changes in marker expression between CB-MSC at passage 2, 3 and 5 were noted for CD90 (p = 0.50 (passage 2 to 3), p = 0.60 (passage 3 to 5)). Low/negative surface expression of other markers on CB-MSC precluded relevant statistical analysis. In WBC, two populations of cells with distinct CD90 expression were detected, possibly reflecting lymphocytes and neutrophils, while in CB-MSC CD90 expression was more homogeneous (Fig 2). To confirm the lack of surface expression of MHC-I on CB-MSC at passage 5, MHC-I clone CVS22 was additionally assessed and showed similar lack of expression (data not shown).

**Fig 3:**
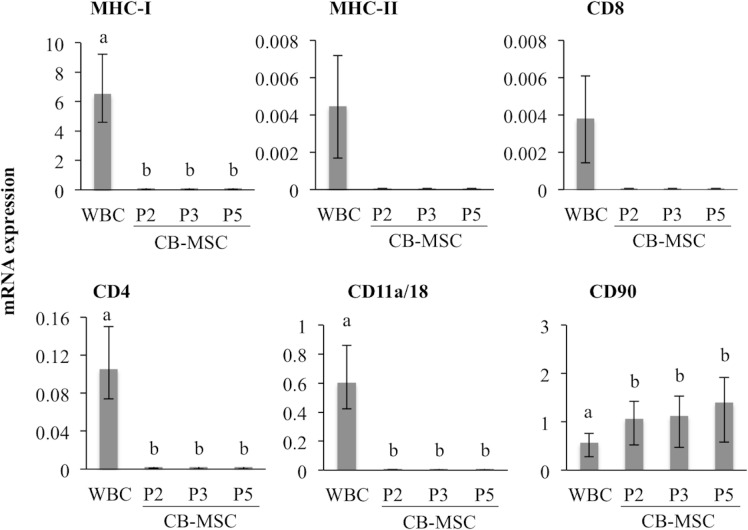
Relative mRNA expression in WBC and CB-MSC. Gene expression were assessed with qPCR and normalized to S18 and β -actin, and is shown relative to the lowest average CT. Bars represent CI; different letters indicate significant differences (*p* < 0.05) between groups. Transcripts for MHC-II and CD8 were detected only in WBC.

Relative quantification of gene transcripts correlated with flow cytometric detection of antigen expression (Fig 3). Transcripts of antigens expressed on WBC were either not detected or extremely low in CB-MSC at each passage. However, relative CD90 transcript abundance was significantly higher in CB-MSC at passage 2 than in WBC (p = 0.04), and remained high throughout CB-MSC culture. Findings were similar for all samples.

### CD29 and CD44 are highly expressed on CB-MSC

**Fig 4:**
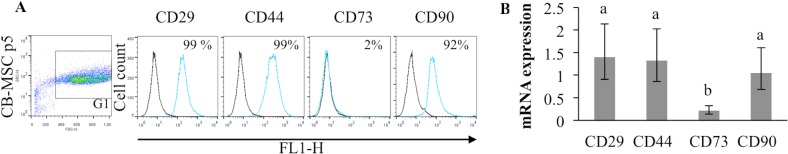
CD29, CD44 and CD90 are highly and consistently expressed on CB-MSC. A. Fluorescence of control samples (black line) and specific antibody (light blue/gray) with the mean proportion of MSC expressing each antigen. Results are representative of 5 experiments. B. Relative mRNA expression. Bars represent CI, and different letters indicate significant differences (*p* < 0.05) between groups. CB-MSC had consistently high expression of CD29, CD44 and CD90 relative to CD73.

Expression of CD29, CD44 and CD73 was assessed on passage 5 CB-MSC. Flow cytometric analysis indicated CD29 and CD44 were expressed on ≥99% of CB-MSC, which was a significantly greater proportion of CB-MSC than those expressing CD90 (p < 0.0001) (Fig 4A). CD73 was either very low or undetectable on CB-MSC. Analysis of mRNA expression of each of these genes also indicated high abundance of CD29, CD44 and CD90 mRNA, and relatively low expression of CD73 mRNA (Fig 4B).

### CB-MSC decrease lymphocyte proliferation in mixed lymphocyte reactions

**Fig 5:**
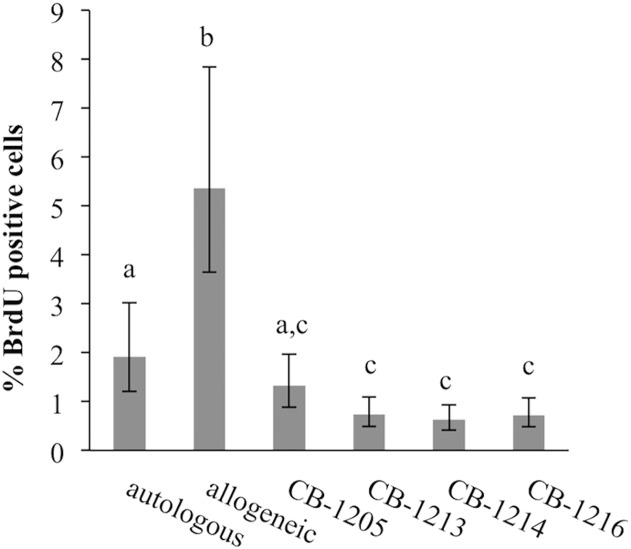
CB-MSC decrease proliferation in mixed lymphocyte reactions. Autologous lymphocytes had minimal proliferation, while culture of allogeneic lymphocytes resulted in proliferation. Addition of CB-MSC to allogeneic lymphocyte cultures reduced proliferation as detected by BrdU incorporation and flow cytometry. Different letters indicate significant differences (*p* < 0.05) between groups, bars indicate CI.

Culture of allogeneic mononuclear cells yielded lymphoproliferation typical of mixed lymphocyte reactions (MLR) while culture of autologous mononuclear cells, as expected, yielded a low proportion of proliferating cells (Fig 5). Addition of CB-MSC reduced allogeneic lymphoproliferation to levels below those of both allogeneic and autologous cells. In all four CB-MSC cultures suppression was significant compared to allogeneic cells (p < 0.0001 for all CB-MSC cultures). In three of four CB-MSC experiments there was significant suppression relative to autologous cells (p = 0.0032 (CB-1213), p = 0.0028 (CB-1216), p = 0.0007 (CB-1216)).

### CB-MSC constitutively express TLR4

**Fig 6:**
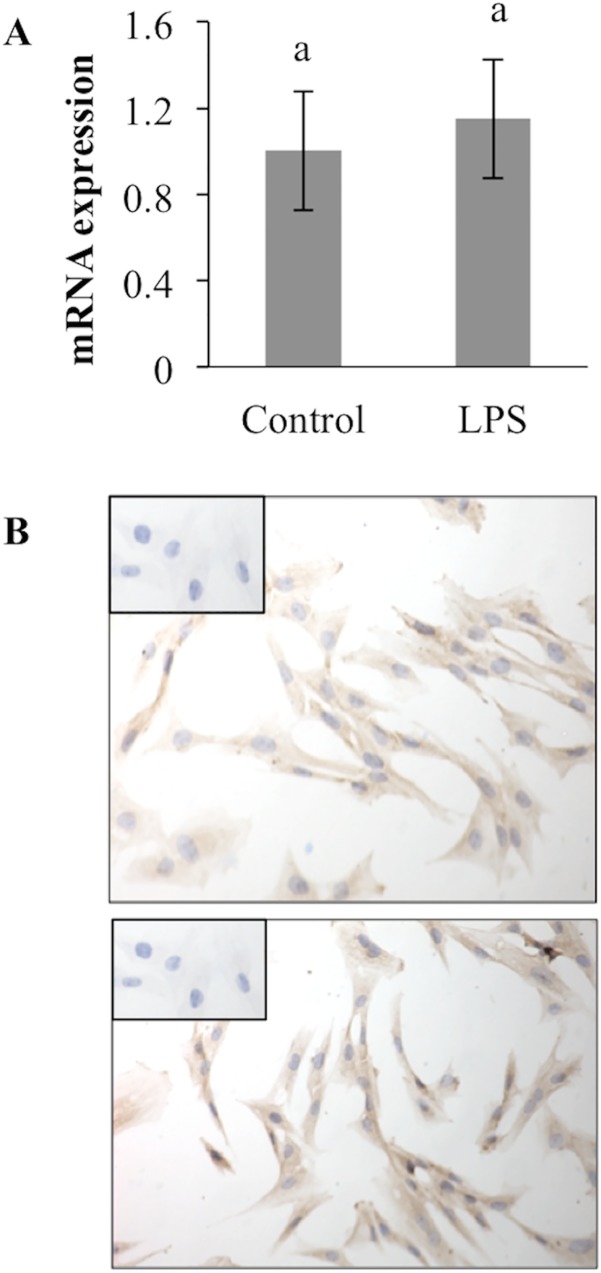
CB-MSC constitutively expresses TLR4 with minimal up-regulation after exposure to LPS. A. TLR4 mRNA was normalized to S18 and β -actin. Bars indicate CI. Different letters indicate significant differences (*p* < 0.05) between groups. B. Untreated (top) and LPS-treated (bottom) CB-MSC have similarly intense TLR4 immunoreactivity. Inset: Omission of primary antibody.

In order to determine possible contributions of TLR3 and TLR4 to functional properties of CB-MSC, expression of each receptor by mRNA quantification and ICC was determined. Neither TLR3 mRNA nor protein was detected by qPCR and ICC assays (data not shown). However, TLR4 mRNA was highly expressed in CB-MSC, and protein was detected in untreated as well as LPS-treated cells (Fig 6). CB-MSC constitutively highly expressed TLR4 mRNA with minimal increase in expression after LPS stimulation.

## Discussion

Equine CB-MSC were derived from each of nine CB samples, suggesting this culture protocol was highly efficient. Although collection of CB is relatively non-invasive and yields highly proliferative MSC with chondrogenic potency [37,45,46], relatively low isolation success has limited their utility to date [47,48]. As a result, CB-MSC are less well characterized relative to BM- and AT-derived MSC. In initial studies of equine CB-MSC, cell lines were derived from 4 of 7 [37], 41 of 51 [49] and 13 of 17 samples [50], e.g. 57–80% isolation frequency. Subsequently, improved culture methods generated MSC from 5 of 5 [38] and 6 of 6 CB samples [12]. In humans, successful derivation of MSC from CB ranges from 29 to 48% irrespective of methodological differences [47,48]. Generation of CB-MSC differs between humans and horses due to the available volume of CB and collection methods. The method described here consistently yielded CB-MSC cultures, even from samples up to 48 hours old. Therefore, in light of definition of robust culture methods, focus may now be placed on timely and consistent collection of CB from mares.

Leukocytes in CB consisted of cells with light scatter properties and antigen expression typical of lymphocytes, neutrophils and monocytes. Derivation of CB-MSC was associated with loss of WBC and appearance of a uniform population of large fibroblast-like cells with very high forward light scatter. CB-MSC were characterized by lack of expression of typical WBC antigens, and by expression of CD29, CD44 and CD90. High expression of CD29, CD44 and CD90 and low expression of CD73 was previously reported for equine MSC [14,16,51]. CD29 and CD44 are molecules that function in cell adhesion, and high expression is associated with stem cell phenotype [52]. CD90 is glycosylated immunoglobulin-family member protein widely expressed on many types of stem cells and WBC [53,54]. CD73 is an ectonuclease and the CD73 (clone 10f1) used in this study was expressed on human and horse WBC, with nucleotide identity of 88% between horses and humans (data no shown). Hence, the antibody utilized was expected to detect equine CD73, which implies that our CB-MSC are indeed low/negative for CD73. In rodents, CD73 expression has been associated with small AT-derived MSC, while large AT-derived MSC lacked CD73 [55]. Expression of CD73 correlated with differentiating properties of MSC. Whether similar functional properties apply to CB-MSC remains to be determined.

Consistent low or absent expression of MHC-I on equine CB-MSC is in contrast to findings by Carrade et al. (2012) and De Schauwer et al. (2014). In those studies, CB-, umbilical cord matrix (UCM)-, peripheral blood (PB)-, BM- and AT-derived MSC expressed MHC-I [12,23]. Lack of MHC-I expression on CB-MSC at passage 5 was also noted with antibody CVS22 as used by Carrade et al. (2012) (data not shown). Lack of detection of MHC-I with different antibodies by flow cytometry, and lack of mRNA detection, indicate that CB-MSC derived under conditions described here do not express MHC-I. Lack of MHC-I and-II expression may contribute to or account for lymphosuppressive properties of MSC during co-culture with mononuclear cells from a range of unrelated donors [56]. Nevertheless, since expression of MHC-I and-II on MSC has been suggested to be unstable [23,32,57], transient downregulation during the first 5 passages cannot be ruled out. Additional investigations on stability and mechanisms of MHC regulation in MSC are needed. Resolving presence or absence of highly polymorphic molecules such as MHC may aid in their potential therapeutic application in allogeneic patients.

Equine CB-MSC suppressed proliferation of allogeneically stimulated lymphocytes in co-culture experiments. Carrade et al. (2012) reported similar findings for CB and other tissue-derived MSC. However, results from *in vivo* studies are controversial. MHC compatibility and MSC concentration may influence the suppressive effect of MSC [58]. Heterogeneous ability of MSC to suppress immune response was also suggested to account for lack of immunosuppressive effect in recent clinical trials [59]. Immunomodulatory mechanisms of MSC are poorly understood and antigens predictive of lymphosuppressive ability have not been identified, therefore, such functions cannot be ascribed to specific cells within a heterogeneous culture of MSC. Human pro-inflammatory and anti-inflammatory macrophages have discrete antigen expression and cytokine production [60]. Among these, expression of TLR4 was suggested to correspond to an anti-inflammatory or immunosuppressive phenotype [33]. Based on reports of plasticity of TLRs [33,61], up-regulation of TLR3 and TLR4 following stimulation with poly (I:C) and LPS, respectively, was expected. However, TLR4 mRNA and protein were constitutively expressed in untreated CB-MSC, and treatment with LPS upregulated expression only slightly. Constitutive expression of TLR3 mRNA or protein was undetectable or minimal in CB-MSC (data not shown). These findings suggest that CB-MSC derived under conditions employed here have distinct expression of TLR4 but not TLR3, which may correspond to their ability to suppress allogeneic lymphoproliferation. Future studies need to be directed at evaluating different culture conditions and multiple time points following exposure to TLR ligands, and at investigating other molecules that may convey immunomodulatory function [33,61]. Furthermore, TLR3 and TLR4 may not respond to identical stimuli in different species, and more extensive optimization of protocols for equine cells may be required [62,63].

## Conclusion

A protocol for consistent generation of MSC from equine CB is described. Resulting CB-MSC have a cell antigen phenotype that is cryotolerant and consists of high expression of CD29, CD44 and CD90, and low or absent expression of MHC-I, MHC-II, CD4, CD8, CD11a/18 and CD73. CB-MSC constitutively expressed TLR4 but not TLR3, and suppressed lymphocyte proliferation in allogeneic co-cultures. These findings comprise an important advance in derivation of CB-MSC for clinical use.
